# Return to work after traumatic spinal fractures and spinal cord injuries: a retrospective cohort study

**DOI:** 10.1038/s41598-023-50033-3

**Published:** 2023-12-19

**Authors:** Fateme Keihanian, Enayatollah Homaie Rad, Simin Samadi Shal, Nooshin Pourreza, Leila Khoochakinejad Eramsadati, Seyedeh Mitra Hosseini Malekroudi, Naema Khodadadi-Hassankiadeh

**Affiliations:** 1 Guilan Road Trauma Research Center, Trauma Institute, Guilan University of Medical Sciences, Rasht, Iran; 2 Social Determinants of Health Research Center, Trauma Institute, Guilan University of Medical Sciences, Rasht, Iran; 3 School of Medicine, Guilan University of Medical Sciences, Rasht, Iran; 4 Erasmus MC University Medical Center, Rotterdam, The Netherlands; 5 Guilan Road Trauma Research Center, Poursina Hospital, Trauma Institute, Guilan University of Medical Sciences, Namjoo St, Rasht, Iran

**Keywords:** Diseases, Trauma, Neuroscience, Spinal cord injury

## Abstract

This study aimed to determine the factors associated with return to work (RTW) after traumatic spinal fracture and spinal cord injury. It provided a predictive model for RTW among patients with spinal fractures and spinal cord injury and determined important factors influencing the time to RTW after injury. A retrospective cohort study was conducted in Poursina Tertiary Hospital, Guilan, Iran between May 2017 and May 2020. Patients aged 18 to 65 who were hospitalized with traumatic spinal fractures and spinal cord injuries were included. Demographic and clinical data were collected from the National Spinal Column/Cord Injury Registry of Iran (NSCIR-IR). A researcher-administered questionnaire was used through a telephone interview to obtain complementary data on social and occupational variables. Kaplan–Meier survival analysis was used to estimate the average time to RTW and the predictors of RTW were determined by multivariate Cox regression model. Of the 300 patients included, 78.6% returned to work and the average time to RTW was about 7 months. The mean age of the participants was 45.63 ± 14.76 years old. Among the study variables, having a Bachelor’s degree (HR 2.59; 95% CI 1.16–5.77; P = 0.019), complications after injury (HR 0.47; 95% CI 0.35–0.62; P = 0.0001), full coverage health insurance (HR 1.73; 95% CI 1.10–2.72; P = 0.016), opium use (HR 0.48; 95% CI 0.26–0.90; P = 0.023), number of vertebral fractures (HR 0.82; 95% CI 0.67–0.99; P = 0.046), and length of hospital stay (HR 0.95; 95% CI 0.93–0.98; P = 0.001) were found to be significant in predicting RTW in Cox regression analysis. Our analysis showed that wealthier people and those with high job mobility returned to work later.

## Introduction

Traumatic spinal fractures (TSFs) and spinal cord injuries (SCIs) are devastating conditions with a high burden of physical, emotional, and economic consequences for patients, families, and society^1^. In the United States, the overall prevalence of spine fracture has been estimated to be 5.4%, increasing with age^2^. In Iran, TSF was reported in 3.8% of trauma admissions between 1999 and 2004 using the National Trauma Registry data, and 5.8% of patients had a concurrent SCI^3^. Although the incidence of SCI has not changed significantly, the total number of patients with SCI is likely to be increasing due to the growth of the world’s population^1^. Worldwide, the average prevalence of SCI is estimated to be 1:1000, and the mean incidence is proposed to be between four and nine cases per 100,000 people per year. It varies substantially in different parts of the world. The mean incidence of SCI in developing countries is about 25.5/million/per year. The incidence of SCI in industrialized countries ranges from 15 in Western Europe to 39/million/year in the USA^4^. The annual prevalence of SCI has been reported to be ten in one million in Tehran, Iran, and more common in men and younger people^5^. In another study in Iran, TSF incidence was 16.35 (95% CI 3.4–48.0) per 100,000, and SCI was found in about half of the TSF patients^6^. The main causes of traumatic SCIs across most geographical regions are falls and road traffic accidents^7^.

The incidence of SCI peaks in young adulthood and, to a lesser extent, in old age. Recent studies showed an increase in age at the time of injury. Men are most at risk in young adulthood (20–29 years) and older age (70+). Women are most at risk in adolescence (15–19) and older age (60+). Studies reported a male-to-female ratio of at least 2:1 among adults^8^. In a meta-analysis, the proportion of cases with traumatic SCI in the 15–29 age group decreased from 50% (1961–1980) to 20% (2001–2020), while it increased from 9 to 35% in the 60+ age group^9^.

Since the spinal cord is the highway that allows the brain to control the rest of the body, SCIs significantly affect the patient’s quality of life^10^. Work participation rates among people with SCI are substantially below that of the general population^11^. The benefits of employment to a person’s physical, psychological, and financial health are numerous and well documented. Employment is associated with increased social integration, better physical and mental health, and improved quality of life^12^. Subsequently, worker disability and absence from the workforce are associated with significantly diminished economic, health, and psychosocial well-being^13^. RTW rates following a disability like SCI are estimated to vary from 11.5 to 74% on a global scale. Several factors are involved including differences in injury compensation, health care and support systems, legislation, as well as methodological and measurement issues^14^. In this study, we aimed to determine the rate of RTW after TSF/SCIs and identify the factors associated with RTW.

## Materials and methods

### Study design and population

This was a retrospective cohort study. The participants were TSF/SCI patients admitted to the Poursina Tertiary Hospital, Guilan Province, Iran from May 2017 to May 2020.

Patients aged between 18 and 65 years (the working-age population), hospitalized with penetrating or blunt trauma to the vertebral column were included in our study. Since January 1, 2016, the data of all TSF/SCI patients admitted to Poursina Hospital has been registered in NSCIR-IR, which is a hospital-based, and prospective observational registry of individuals who sustained TSF/SCIs^15^.

We extracted the patients’ list and their demographic and clinical characteristics from the registry. To obtain data about social and occupational parameters, a telephone interview was conducted with each patient, so that all samples were given the same opportunity to participate in the interview.

The Exclusion criteria were as follows: (1) Patients who did not have a contact number or those who did not answer the phone after three phone calls at random hours during 2 weeks. (2) Patients whose families had reported their deaths. (3) Individuals who were not working at the time of injury (they were students, unemployed, or retired). (4) Those who did not agree to participate in the study.

### Measurement

The data required for this study were collected from two sources: NSCIR-IR and telephone interview data. First, the data of the predetermined variables of NSCIR-IR were extracted. Then, a researcher-administered questionnaire was provided to collect other required data through a telephone interview. The questionnaire was given to 5 neurosurgeons and 5 neurologists to determine its validity, and the suggested amendments were applied to the questionnaire. The reliability of this questionnaire was measured by the method of internal consistency, and Cronbach's alpha coefficient was 71%.

The patients primarily answered the questions in a telephone interview, but if they did not recall the required information, a family member could help. Informed consent was obtained orally from all patients eligible for study participation. Patients’ data were saved and used anonymously.

The data obtained from each source was as follows:

(1) NSCIR-IR database

Demographic factors: age, gender, marital status (single, married, widowed, and divorced), educational level (illiterate, elementary, junior high school, high school, diploma, associate graduate, bachelor, master, and uncertain).

Clinical factors: American Spinal Injury Association Impairment Scale (AIS)^16^, length of hospital stay, length of ICU stay, concomitant injuries (limb fracture or dislocation, burn, internal damage, and brain injury), underlying disease (hypertension, diabetes, hypothyroid, osteoporosis, and rheumatoid arthritis), neurological category (quadriplegia, paraplegia, paraparesis hemiparesis, quadriparesis, and other), number of vertebral fractures, and vertebral fracture site (cervical, thoracic, lumbar, sacral, and coccygeal).

(2) Call interview data

Social factors: sedative use (cigarette, opium, drug), insurance status (without insurance, full coverage insurance, incomplete coverage insurance), Wealth index.

Occupational factors: pre-injury occupation (housewife, daily wage worker, self-employed, government employee, private-sector employee), employment type (part-time/full-time), RTW (yes/no), time to RTW, type of RTW (return to previous job, adjusted RTW, and getting into the new job), reason for not returning to work (pain, early retirement, complications, other), job mobility (no mobility, low mobility, moderate mobility, high mobility).

Wealth index: The wealth index is based on the patient's answers to questions about owning a house and its size, type of heating system, type of television, type of sanitation, access to the smartphone, personal computer, and car, refrigerator, motorbike. Using principal component analysis (PCA), we calculate the wealth index for each household and categorize the wealth into three groups: poor, middle, and rich.

Job mobility was defined as the type of activity that is associated with a job or occupational physical activity.

Time to RTW was defined as the time interval between the injury and the first RTW. The time variable was constructed by subtracting the RTW date provided by the patient from the date of the injury as documented in the registry.

### Statistical analysis

Descriptive statistics such as frequency and percentage, mean and standard deviation were used to describe the research data. The normality of the data distribution was verified using the Kolmogorov–Smirnov test. Based on RTW status, patients were divided into two groups: (1) RTW group and (2) non-RTW (NRTW) group. To compare the quantitative variables of the two groups, Independent *t* test or Mann–Whitney test was used. For the qualitative variables of the two groups, Chi-square or Fisher exact test was applied. The main variable in the analysis was the time to RTW. We performed survival analysis using Kaplan–Meier curves to estimate the average time to RTW for the whole group. Potential predictors of the time to RTW were analyzed with multivariate Cox regression. We entered all data in Stata version 14. A P-value less than 0.05 was considered significant in all tests.

### Ethics declarations

The study was approved by the Human Research Ethics Committee (HREC), Guilan University of medical sciences (IR.GUMS.REC.1399.570). The study was conducted in accordance with the Declaration of Helsinki. Ethical consent was obtained from all participants to participate in the research.

## Results

Data of 392 people with TSF/SCI were extracted from NSCIR-IR, of which 32 patients were excluded due to unemployment at the time of injury, 50 people were not cooperative or did not answer the phone, and 10 died after hospital discharge. Finally, 300 patients were included in the study analysis.

In total, 236 patients (78.66%) returned to work, and the average time to RTW was 6.9 ± 5.2 months. The mean age of the participants was 45.63 ± 14.76 years, and comparing the two groups, the patients in the RTW group were significantly younger (P = 0.007). Most patients were male (73.33%) and married (77%). The most frequent educational level in the RTW group was junior high school (26.3%), and in the NRTW group was diploma (25%), and there was no significant difference in educational level between the two groups (P = 0.44). The majority of participants (66%) had full coverage insurance. For pain relief, 24.66% used drugs. The sociodemographic characteristics of the two groups are summarized in Table 1.

**Table 1:** Comparison of sociodemographic variables in the RTW group and NRTW group.

Characteristics	RTW (n = 236)	NRTW (n = 64)	P-value
Age, (mean ± SD) years	44.28 ± 13.90	50.60 ± 16.79	0.007
Gender, n (%)			0.001
Male	163 (69.1)	57 (89.1)	
Female	73 (30.9)	7 (10.9)	
Marital status, n (%)			0.477
Single	43 (18.2)	11 (17.2)	
Married	179 (75.8)	52 (81.2)	
Widowed	7 (3)	0	
Divorced	7 (3)	1 (1.6)	
Educational level, n (%)			0.446
Illiterate	29 (12.3)	15 (23.4)	
Elementary	43 (18.2)	11 (17.2)	
Junior high school	62 (26.3)	13 (20.3)	
High school	17 (7.2)	4 (6.3)	
Diploma	59 (25)	16 (25)	
Associate graduate	3 (1.3)	2 (3.1)	
Bachelor	20 (8.5)	3 (4.7)	
Master	2 (0.8)	0	
Uncertain	1 (0.4)	0	
Insurance status, n (%)			0.003
Without insurance	25 (10.6)	17 (26.5)	
Full coverage insurance	165 (69.9)	33 (51.6)	
Incomplete coverage insurance	46 (19.5)	14 (21.9)	
Sedative use, n (%)			0.001
None	119 (50.4)	22 (34.4)	
Cigarettes	55 (23.3)	8 (12.5)	
Opium	11 (4.7)	11 (17.2)	
Drug	51 (21.6)	23 (35.9)	

The majority of the studied population was self-employed (52.33%), and 85% had full-time employment. Of the total subjects, 52.66% had returned to their previous jobs, and 21.33% had adjusted RTW. The reasons for not returning to work in 51.66% of cases were pain, 39% were post-injury complications, 2.3% were early retirement, and 7% were other causes. More than half of the patients (51.33%) had work with moderate mobility. The occupational characteristics of the participants according to RTW status are presented in Table 2.

**Table 2:** Comparison of occupational variables in the RTW group and NRTW group.

Characteristics	RTW (n = 236)	NRTW (n = 64)	P-value
Type of RTW, n (%)			0.001
Return to previous job	158 (66.9)	–	
Adjusted RTW	64 (27.1)	–	
Back to the new job	14 (5.9)	–	
Work mobility, n (%)			0.001
No mobility	1 (0.4)	0	
Low mobility	27 (11.4)	4 (6.2)	
Moderate mobility	136 (57.6)	18 (28.1)	
High mobility	72 (30.5)	42 (65.6)	
Pre-injury occupation, n (%)			0.004
Housewife	60 (25.4)	5 (7.8)	
Daily wage worker	21 (8.9)	13 (20.3)	
Self-employed	120 (50.8)	37 (57.8)	
Government employee	14 (5.9)	6 (9.4)	
Private employee	21 (8.9)	3 (4.7)	
Employment type, n (%)			0.005
Part-time	28 (11.9)	17 (26.6)	
Full-time	208 (88.1)	47 (73.4)	

### Clinical findings

The vast majority of subjects were classified AIS E (95.8% in the RTW group vs. 82.8% in the NRTW group), and among SCI patients, AIS A was the most common classification. Most patients with TSFs did not have neurologic impairments and were considered neurologically intact. Among SCI patients, paraplegia was the most common neurological category. The most common concomitant injury was limb fracture and dislocation (18.2% in the RTW group vs. 31.3% in the NRTW group, P = 0.020).

The most prevalent underlying disease was hypertension (7.2% in the RTW group vs 15.6% in the NRTW group, P = 0.040). In terms of the number of vertebral fractures, the majority of participants had a single fractured vertebra (68.6% in the RTW group vs. 48.4% in the NRTW group), and the difference between the two groups was significant (P = 0.004). The most common vertebral fractures were lumbar fractures in total subjects (53.33%). There was a significant difference between the two groups in the fractures of thoracic vertebrae (P = 0.034) (Table 3).

**Table 3:** Comparison of clinical characteristics in the RTW group and NRTW group.

Clinical characteristics	RTW (n = 236)	NRTW (n = 64)	P-value
Length of hospital stay, median (range) day	4 (0–40)	6.5 (1–26)	0.001
Length of ICU stay, median (range) day	1 (1–44)	3 (1–36)	0.223
AIS, n (%)			
A	5 (2.1)	9 (14.1)	0.001
B	2 (0.8)	0	
C	2 (0.8)	0	
D	1 (0.4)	2 (3.1)	
E	226 (95.8)	53 (82.8)	
Neurological category, n (%)			
Paraplegia	7 (2.9)	2 (3.1)	0.040
Quadriplegia	3 (1.3)	4 (6.3)	
Paraparesis	2 (0.85)	0	
Hemiparesis	0	1 (1.6)	
Quadriparesis	1 (0.4)	0	
Others	3 (1.3)	3 (4.7)	
Concomitant injury, n (%)			
Limb fracture or dislocation	43 (18.2)	20 (31.3)	0.020
Burn	1 (0.4)	0	0.787
Internal damage	5 (2.1)	0	0.299
Brain injury	8 (3.4)	7 (10.9)	0.022
Underlying disease, n (%)			
Hypertension	17 (7.2)	10 (15.6)	0.040
Diabetes	13 (5.5)	7 (10.9)	0.101
Hypothyroid	1 (0.4)	0	0.787
Osteoporosis	0	1 (1.6)	0.213
Rheumatoid arthritis	2 (0.8)	0	0.618
Number of fractured vertebra, n (%)			
1	162 (68.6)	31 (48.4)	0.004
2	60 (25.4)	24 (37.5)	
3	8 (3.4)	3 (4.7)	
4	2 (0.8)	4 (6.3)	
5	0	1 (1.6)	
6	2 (0.8)	1 (1.6)	
Missing	2 (0.8)	0	
Vertebral fracture site, n (%)			
Cervical	74 (31.4)	15 (23.4)	0.141
Thoracic	62 (26.3)	25 (39.1)	0.034
Lumbar	125 (53)	35 (54.7)	0.459
Sacral	0	0	–
Coccygeal	0	0	–

### Predictors of RTW

Multivariate Cox regression models were applied to find the factors that significantly affect the time to RTW. Among the study variables, having a Bachelor’s degree (HR 2.59; 95% CI 1.16–5.77; P = 0.019), complications after injury (HR 0.47; 95% CI 0.35–0.62; P = 0.0001), full coverage health insurance (HR 1.73; 95% CI 1.10–2.72; P = 0.016), opium use (HR 0.48; 95% CI 0.26–0.90; P = 0.023), number of vertebral fractures (HR 0.82; 95% CI 0.67–0.99; P = 0.046), and length of hospital stay (HR 0.95; 95% CI 0.93–0.98; P = 0.001) were found to be significant in predicting RTW in Cox regression analysis (Table 4).

**Table 4:** Multivariate Cox regression of RTW predictors in patients with TSF/SCI.

Variable	HR	SE	P-value	95% CI
Education level				
Illiterate	1.00			
Elementary	1.20	0.33	0.49	0.70–2.07
Junior high school	1.40	0.36	0.195	0.83–2.35
High school	1.07	0.38	0.838	0.53–2.16
Diploma	1.35	0.39	0.299	0.76–2.41
Associate graduate	1.31	0.89	0.689	0.34–4.97
Bachelor	2.59	1.05	0.019	1.16–5.77
Master	0.50	0.39	0.388	0.10–2.38
Uncertain	0.62	0.64	0.651	0.82–4.76
Reason of not returning to work				
Pain	1.00			
Early retirement	0.19	0.19	0.108	0.02–1.42
Complications	0.47	0.06	0.000	0.35–0.62
Others	1.31	0.31	0.240	0.83–2.09
Insurance status				
Without insurance	1.00			
Full coverage insurance	1.73	0.39	0.016	1.10–2.72
Incomplete coverage insurance	1.53	0.39	0.097	0.92–2.53
Sedative use				
None	1.00			
Cigarette	0.83	0.13	0.267	0.60–1.14
Opium	0.48	0.15	0.023	0.26–0.90
Drug	0.82	0.13	0.244	0.59–1.14
Number of vertebral fracture	0.82	0.08	0.046	0.67–0.99
Length of hospital stay	0.95	0.01	0.001	0.93–0.98

*HR* hazard ratio, *SE* standard error, *CI* confidence interval.

**Figure 1:**
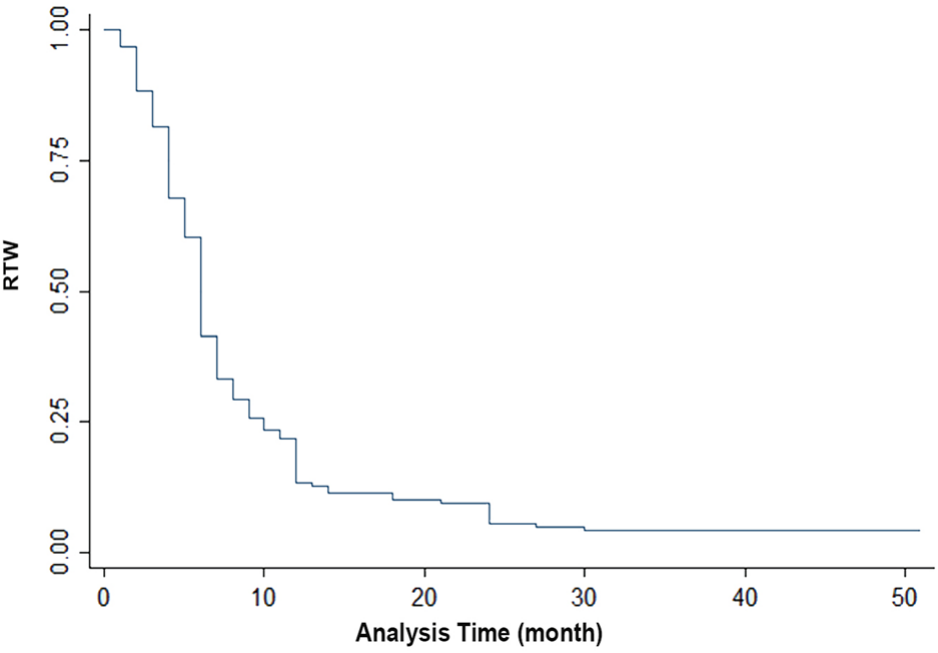
Kaplan–Meier survival curve showing the average time of RTW.

Kaplan–Meier survival curve demonstrated the average time to RTW, with 75% of patients returning to work within the first 10 months and approximately 90% within the first 25 months (Fig. 1).

**Figure 2:**
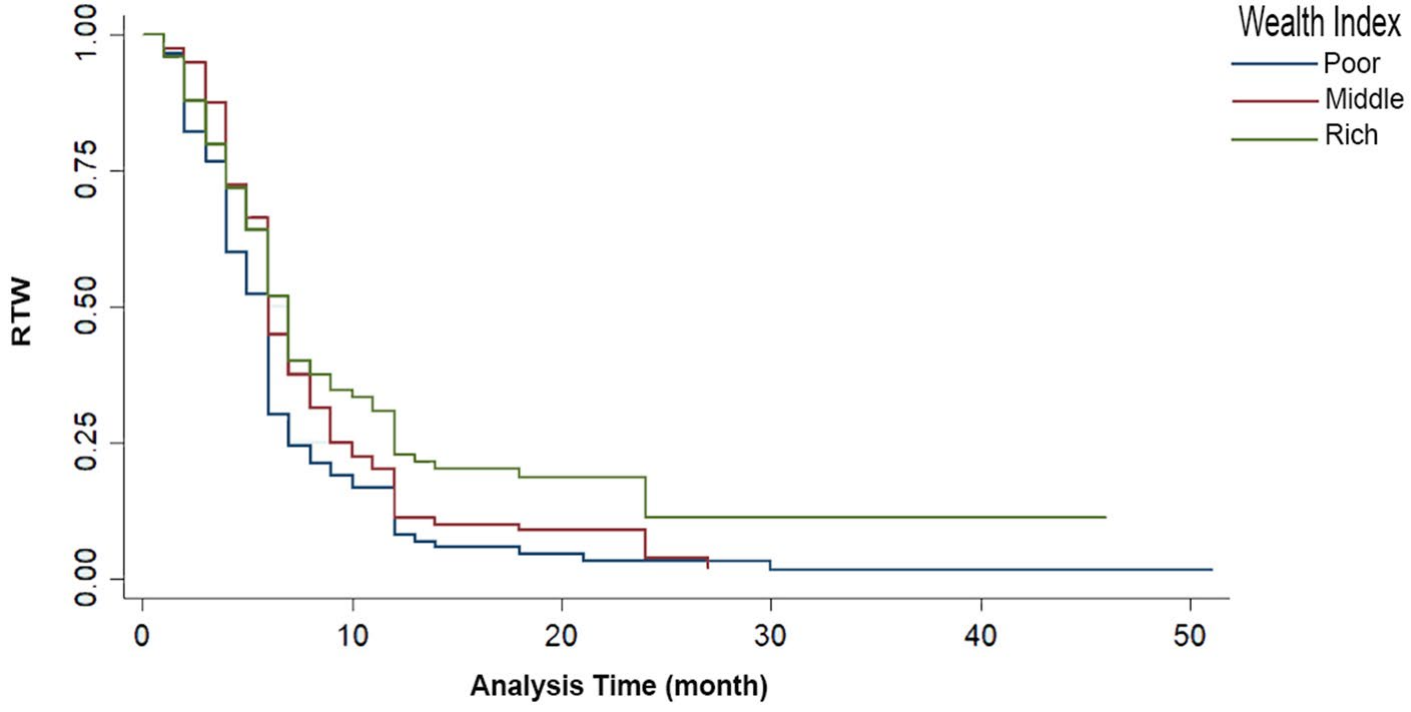
Kaplan–Meier survival curve indicating the correlation between RTW and wealth index.

Kaplan–Meier survival estimates indicated that wealthier people returned to work later (Fig. 2).

**Figure 3:**
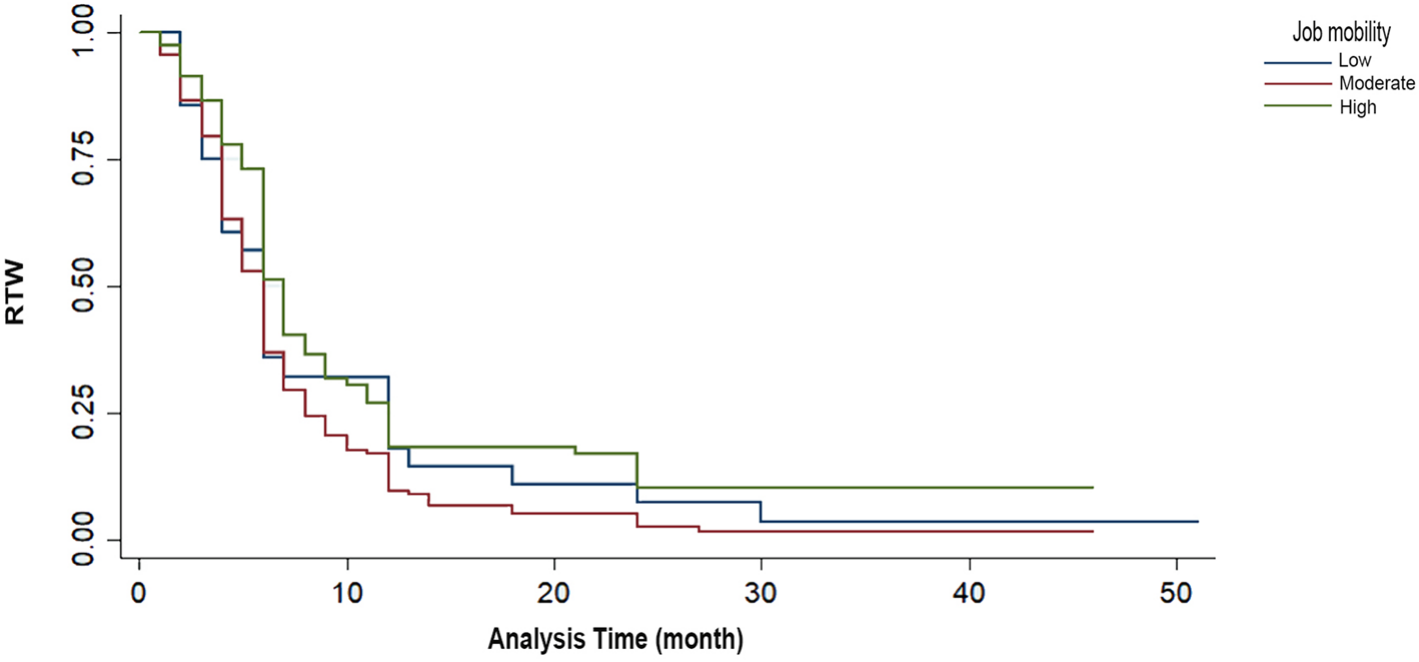
Kaplan–Meier survival curve indicating the correlation between RTW and job mobility.

Finally, the Kaplan–Meier survival estimates suggested that people with high job mobility returned to work later (Fig. 3).

## Discussion

In our study, approximately 79% of TSF/SCI patients returned to work in a mean time of about 7 months. Similarly, a systematic review by Lidal et al. indicated that the employment rate of patients after SCI ranged from 11.5 to 74% in different countries^17^. Compared with the results of similar studies in Germany (RTW rate: 42.5%)^18^, Switzerland (RTW rate: 53.4%)^19^, and Taiwan (RTW rate: 30.3%)^20^, the rate of RTW in our study was in the high range. Among Iranian studies, the RTW rates after trauma were reported 61.2% at 3 months post-injury^21^ and 75.3% at 1 year^22^. Since our study comprised TSF patients with or without SCI, and most patients with TSF did not have considerable neurological impairment, the high rate of RTW in the present study seems reasonable.

The results in the current paper showed that a bachelor’s degree had a significant relation with earlier RTW. This is almost congruent with the findings of the previous studies reporting educational level as a strong predictor of RTW, and higher levels of education were positively correlated to RTW^23–25^. The possibility of re-employment increases with educational level and education is a facilitating factor^18,26^. Patients with higher levels of education are typically employed in less physically demanding jobs and may have access to more flexible employment opportunities. They have more personal motivation and expectations. Furthermore, higher education is associated with health-promoting behavior and improves the outcome of rehabilitation programs^27^.

In the present study, the longer length of hospitalization had a significant relationship with delayed RTW. Previous studies have also shown that the duration of hospitalization can be a good indicator of the severity of injuries and can cause a delay in returning to work^28,29^. Consistent with our findings, Abedzadeh-Kalahroudi et al. reported that the rate and time of RTW among trauma patients with longer hospital stays were respectively higher and longer^21^.

Our findings indicated that the number of vertebral fractures was negatively correlated with the time to RTW. Fractures often have a longer healing process, followed by waiting for surgery and longer hospital stays. Therefore, a slower return to normal function and work is expected^30,31^.

According to the Cox regression model, there was a strong correlation between post-injury complications and time to RTW, and those with complications returned to work later. Similarly, in one study, despite good surgical results, patients with higher back pain and lower ability index with longer duration of symptoms returned to work later^32^.

The findings of the present study demonstrated that individuals with full-coverage health insurance returned to work earlier. The role of health insurance is potentially noteworthy, as it can influence the process of post-injury treatment. Full-coverage health insurance can be associated with more use of health care and rehabilitation services. In a survival analysis, longer RTW survival rates were found among patients without insurance coverage, and those with insurance were more likely to return to their jobs^21^. Many people in Iran obtain insurance through their employers. Maintaining current insurance and the fear of losing insurance benefits can provide a considerable incentive for returning to work or looking for a job.

Among sedatives, using opium was a negative predictor for returning to work, and opium users took longer to RTW. A possible explanation is that opium users usually lack job stability and may lose their jobs due to dysfunction and dependence. In a study by Abedzadeh-Kalahroudi et al., RTW time was significantly longer among drug abusers than non-abusers. However, in a multivariate analysis adjusting for confounders, drug abuse was not detected as a predictor of RTW^21^.

In the present study, job mobility was not a prognostic factor for returning to work, but people with high job mobility returned to work later. In a similar study, functional independence was a strong predictor of RTW, and it was reported that work environment modifications could improve employability after SCI. In addition, those whose previous jobs were manual had a better RTW^33^. The SCI population was reported to require more assistance or intervention regarding RTW. Unmet needs and workplace issues expressed by employed people identify gaps in RTW and job satisfaction that can affect employment sustainability that need to be addressed^34^.

Kaplan–Meier estimates showed that wealthier people returned to work later, although it was not a significant predictor. In another study, there was no significant difference in the comparative incomes of patients with different estimated RTW outcomes^35^. Financial issues are a strong incentive for returning to work. A higher wealth index leads a person to RTW later and rest more due to the possibility of paying better living expenses^36^. Conversely, in a study that investigated the role of insurance and income on RTW after SCI, wealthier patients returned to work earlier. A possible reason explained was the ability to purchase equipment and safe transportation and hire an assistant^37^.

Although this study had a good sample size and the data was obtained over a long period of 3 years, there are some limitations. We conducted a single-center, not population-based study with retrospective data collection so that the results must be interpreted and generalized with caution. In addition, the time of RTW was self-reported, with the potential for retrospective recall bias. Returning to work is a dynamic and multifactorial process. In this study, we investigated some sociodemographic, and clinical aspects; it is recommended that future studies examine other potentially effective factors in a larger-scale and multi-center design.

## Conclusion

Returning to work after TSF/SCI is a challenging and multifaceted issue. There are wide-ranging benefits to regaining and maintaining employment for people following spinal injuries, including financial benefits, having social contacts, life satisfaction, and a sense of purpose. Our study revealed the correlation between different factors and RTW. Possible predictors of RTW in our study were pre-injury educational level, length of hospital stay, number of vertebral fractures, using opium, having full-coverage health insurance, and post-injury complications. Identifying the modifiable factors associated with RTW can help rehabilitation professionals and health policymakers plan appropriate interventions to improve the employment status of these patients.

## Data Availability

The data that support the findings of this study are available from the corresponding author upon reasonable request.

## References

[1] Badhiwala JH, Wilson JR, Fehlings MG (2019). Global burden of traumatic brain and spinal cord injury. Lancet Neurol..

[2] Cosman F (2017). Spine fracture prevalence in a nationally representative sample of US women and men aged ≥40 years: Results from the National Health and Nutrition Examination Survey (NHANES) 2013–2014. Osteoporos. Int..

[3] Heidari P (2010). Spinal fractures resulting from traumatic injuries. Chin. J. Traumatol..

[4] Thietje R, Hirschfeld S (2017). Epidemiology of spinal cord injury. Neurological Aspects of Spinal Cord Injury.

[5] Sharif-Alhoseini M, Rahimi-Movaghar V (2014). Hospital-based incidence of traumatic spinal cord injury in Tehran, Iran. Iran. J. Public Health.

[6] Moradi-Lakeh M (2011). Burden of traumatic spine fractures in Tehran, Iran. BMC Public Health.

[7] Ottomanelli L, Lind L (2009). Review of critical factors related to employment after spinal cord injury: Implications for research and vocational services. J. Spinal Cord Med..

[8] Bickenbach J (2013). International Perspectives on Spinal Cord Injury.

[9] Moschovou M (2022). Temporal changes in demographic and injury characteristics of traumatic spinal cord injuries in Nordic countries—A systematic review with meta-analysis. Spinal Cord.

[10] Ahmed A, Patil AA, Agrawal DK (2018). Immunobiology of spinal cord injuries and potential therapeutic approaches. Mol. Cell. Biochem..

[11] Post, M. W., Reinhardt, J. D. & Escorpizo, R. Return to work after spinal cord injury*.* In *Handbook of Disability, Work and Health*, 417–429 (2020).

[12] Murphy GC (2009). Putting a vocational focus back into rehabilitation. Aust. J. Career Dev..

[13] Bloom J, Dorsett P, McLennan V (2017). Integrated services and early intervention in the vocational rehabilitation of people with spinal cord injuries. Spinal Cord Ser. Cases.

[14] Ullah MM, Fossey E, Stuckey R (2018). The meaning of work after spinal cord injury: A scoping review. Spinal Cord.

[15] Sharif-Alhoseini M (2019). National Spinal Cord Injury Registry of Iran (NSCIR-IR)—A critical appraisal of its strengths and weaknesses. Chin. J. Traumatol..

[16] Roberts TT, Leonard GR, Cepela DJ (2017). Classifications In Brief: American Spinal Injury Association (ASIA) Impairment Scale. Clin. Orthop. Relat. Res..

[17] Lidal IB, Huynh TK, Biering-Sørensen F (2007). Return to work following spinal cord injury: A review. Disabil. Rehabil..

[18] Sturm C (2020). Promoting factors and barriers to participation in working life for people with spinal cord injury. J. Occup. Med. Toxicol. (London, England).

[19] Reinhardt JD (2016). Labor market integration of people with disabilities: Results from the Swiss spinal cord injury cohort study. PLoS One.

[20] Huang IC (2017). Employment outcomes following spinal cord injury in Taiwan. Int. J. Rehabil. Res..

[21] Abedzadeh-Kalahroudi M (2017). Return to work after trauma: A survival analysis. Chin. J. Traumatol..

[22] Marom BS (2019). Return-to-work barriers among manual workers after hand injuries: 1-year follow-up cohort study. Arch. Phys. Med. Rehabil..

[23] Cancelliere C (2016). Factors affecting return to work after injury or illness: Best evidence synthesis of systematic reviews. Chiropr. Man Ther..

[24] Franceschini M (2012). Occurrence and predictors of employment after traumatic spinal cord injury: The GISEM Study. Spinal Cord.

[25] Etuknwa A, Daniels K, Eib C (2019). Sustainable return to work: A systematic review focusing on personal and social factors. J. Occup. Rehabil..

[26] O’Neill J, Dyson-Hudson TA (2020). Employment after spinal cord injury. Curr. Phys. Med. Rehabil. Rep..

[27] Conti A (2020). Barriers and facilitators of education provided during rehabilitation of people with spinal cord injuries: A qualitative description. PLoS One.

[28] Homaie Rad E (2021). Time of return to work and associated factors in rib fracture victims. Arch. Trauma Res..

[29] Doan HTN (2020). Functional status, pain and return to work of injured motorcyclists involved in a motorcycle crash over one-year post-injury in Vietnam. Injury.

[30] Yang Z (2020). Is hip fracture surgery safe for patients on antiplatelet drugs and is it necessary to delay surgery? A systematic review and meta-analysis. J. Orthop. Surg. Res..

[31] Seong YJ (2020). Timing of hip-fracture surgery in elderly patients: Literature review and recommendations. Hip Pelvis.

[32] Khan I (2019). Impact of occupational characteristics on return to work for employed patients after elective lumbar spine surgery. Spine J..

[33] Jang Y, Wang Y-H, Wang J-D (2005). Return to work after spinal cord injury in Taiwan: The contribution of functional independence. Arch. Phys. Med. Rehabil..

[34] Borg SJ (2022). Factors related to engagement in employment after spinal cord injury in Australia: A cross-sectional study. Arch. Phys. Med. Rehabil..

[35] Folkard SS (2016). Factors affecting planned return to work after trauma: A prospective descriptive qualitative and quantitative study. Injury.

[36] Libeson L (2020). The experience of return to work in individuals with traumatic brain injury (TBI): A qualitative study. Neuropsychol. Rehabil..

[37] Phillips VL, Hunsaker AE, Florence CS (2012). Return to work and productive activities following a spinal cord injury: The role of income and insurance. Spinal Cord.

